# A Clinical Study of the Effectiveness of Two Different 10% Carbamide Peroxide Bleaching Products: A 6-Month Followup

**DOI:** 10.1155/2011/167525

**Published:** 2011-05-05

**Authors:** S. R. Grobler, A. Majeed, R. Hayward, R. J. Rossouw, M. H. Moola, T. J. van W. Kotze

**Affiliations:** Oral and Dental Research Institute, Faculty of Dentistry, University of the Western Cape, Private Bag X1, Tygerberg 7505, Cape Town, South Africa

## Abstract

The purpose of this study was to evaluate the efficacy of two different 10% carbamide peroxide bleaching products just after treatment and after a 6-month follow-up period. *Methods*. Two 10% carbamide peroxide products (Opalescence PF and Nite White ACP) were applied nightly for 14 days, according to the manufacturers' instructions. The color of teeth 11 and 21 of thirty-four subjects having A2 or darker teeth were measured with a spectrophotometer (*L* ^∗^; *a* ^∗^; *b* ^∗^) before treatment, just after treatment (14 days) and after 6 months. *Results and Conclusions*. Both products produced significant whitening of teeth with total color change (Δ*E*_*ab*_^*∗*^) of approximately 5.20 units. There was a significant improvement in all 3 color coordinates (L*, a*, and *b* ^∗^) for up to 6 months postbleaching (*P* < .05). Nite White showed a higher degree of relapse (27%) than Opalescence (18%) over the 6-month period. It is suggested that rebleaching after 6 months is not necessary.

## 1. Introduction

The demand for tooth whitening has increased dramatically over the past years with the resulting development of many new whitening products from as many different companies [1–3]. It seems that the success of tooth whitening depends mainly on the combination of the peroxide concentration and the application period. The way of tooth whitening varies from in-office power bleaching with a higher concentration of peroxide for a short period, to at-home bleaching with a much lower concentration of peroxide but over a longer period. However, the most used peroxide concentration is still believed to be 10% carbamide peroxide because of its low cost, efficacy, ease of use, and safety [3–6].

Many clinical studies [5, 7–12] on various 10% carbamide peroxide products revealed good tooth whitening results which are claimed to last for years [13, 14]. However, the whitening effect shows some relapse in color after the cessation of active bleaching treatment. In a clinical study, Matis et al. [15] reported a significant whitening of teeth following at-home bleaching with 10% carbamide peroxide for 14 days. The average relapse in whitening effect was 45% after 6 months, but the teeth were still significantly whiter as compared to the baseline (prebleaching). A meta-analysis of the clinical trials from 1989 to 1999 on dentist-supervised home bleaching products using 10% carbamide peroxide suggested that only 73% of the bleached population will show a color change of two units or greater, and 50% will require retreatment to maintain this effect for longer than 6 months [16]. 

When studying the articles on the whitening effect of different 10% carbamide peroxide tooth-whitening agents, it became clear that one needs to make groupings so as to be able to compare results. The major differences were found in the application period, the number of applications as well as the original selection of teeth to be whitened as far as their darkness/lightness is concerned. Furthermore, in most cases the recommendations of the manufacturers are not clear. For example, overnight or nightly could be differently interpreted, from a couple of hours/night (3.6 h) [13] up to 11 hours/night [17]. 

Most of the clinical studies evaluated the efficacy of tooth-whitening using dental shade guides. It is a highly subjective method [18] and variables such as observer's experience, eye fatigue, ambient light conditions, and the background against which a tooth is compared may lead to inconsistencies [19]. To overcome these problems, spectrophotometric assessment of tooth shade has been recommended [20, 21].

Over many years, the whitening agent Nite White came through a series of developments (Nite White Excel-2, Nite White Excel-3, Nite White Excel Turbo, etc.), until Nite White ACP 10% carbamide peroxide with the patented amorphous calcium phosphate (ACP) was recently introduced. According to the manufacturers, different compounds such as fluoride, potassium nitrate, and amorphous calcium phosphate (ACP) were included in bleaching products to prevent or lesson demineralization or to stimulate remineralization, so as to prevent tooth sensitivity [22, 23].

From the above, it became clear that there is a major lack of knowledge about the time period necessary until rebleaching should be done as well as what color changes took place during the bleaching process of different bleaching agents.

Therefore, the aim of this study was to compare the effectiveness of two different 10% carbamide peroxide tooth bleaching agents and the relapse in color over a 6-month period.

## 2. Materials and Methods

### 2.1. Bleaching Procedure

This study compared the effectiveness of two 10% carbamide peroxide tray-based home bleaching systems: Opalescence PF 10%, carbamide peroxide, with potassium nitrate and sodium fluoride (Ultradent Products, Inc., South Jordan UT, USA) and Nite White ACP containing 10% carbamide peroxide with potassium nitrate, amorphous calcium phosphate, and fluoride (Discuss Dental, Culver City, CA, USA). 

Thirty-four volunteers willing to have their teeth whitened were randomly divided into 2 groups (2 × 17) for this study. Subjects with generalized tooth sensitivity, poor oral hygiene, presence of fluorosis, or tetracycline staining, smokers, previous use of bleaching products, and pregnant or lactating women were excluded. Only subjects 18 years of age or above with tooth color A2 or darker were included. All participants included in the study signed an informed consent form after full explanation of the project. The study was approved by the Ethics Committee of the University of the Western Cape.

To remove possible extrinsic stains, a dental prophylaxis (Nupro Supreme, Dentsply Int, York, PA, USA) was performed on the anterior teeth at least two weeks prior to the start of the bleaching treatment. 

At the initial examination visit, alginate impressions were recorded and models were poured in yellow stone. Study models were trimmed, and the maxillary bleaching trays were fabricated from 1 mm soft tray material (Discuss Dental, Culver City, CA, USA) using a vacuum forming technique. In the Opalescence group, a light-cured resin block-out material (Ultradent Products, Inc., South Jordan, UT, USA) was used to create the reservoir for the bleaching material while the labial surfaces of teeth on the models were not blocked for treatment in the Nite White group. Trays were trimmed on the labial and lingual surfaces incisal to the free gingival margin, creating a scalloped pattern. 

The two different bleaching products (Nite White ACP and Opalescence PF) were administered overnight (~7 hours) for 14 days using the customized bleaching trays. Participants were instructed to brush twice daily with the toothpaste provided to standardize the fluoride levels and oral hygiene. The treatment process was according to the manufacturer's instructions for both tooth-whitening products. All participants were given verbal and written instructions about the use of their bleaching material. 

The color of teeth 11 and 21 of the subjects was measured with a spectrophotometer (Model: CM-2600d, Konica Minolta Sensing, Inc., Japan) set on the CIE L*a*b* color space. This was done with a 6 mm diameter probe at baseline, immediately after treatment (14 days) and after 6 months. Before use, the instrument was calibrated as outlined by the manufacturer. Three measurements of one area (6 mm diameter) at the center of the crown of the two maxillary central incisors were performed. The average of the three values was recorded as the measured value for statistical analysis.

### 2.2. Statistical Analysis

The data were analyzed using a statistical software package NCSS 2007 (NCSS, LLC, Kaysville, UT, USA). Pre- and postbleaching L*, a*, and b* values were compared using the Wilcoxon Signed Rank Sum Test significant at *P* < .05. The color changes in individual components (ΔL*, Δa* and Δb*) between pre- and postbleaching time intervals were calculated. The whitening effect or color improvement was represented by a positive ΔL* (increased lightness/brightness) and negative Δb* (reduction in yellowness). The total color change Δ*E*_ab_* = [(ΔL*)^2^+(Δa*)^2^+(Δb*)^2^]^1/2^ for both groups was compared using the Wilcoxon Rank Sum Test significant at *P* < .05.

## 3. Results

**Figure 1:**
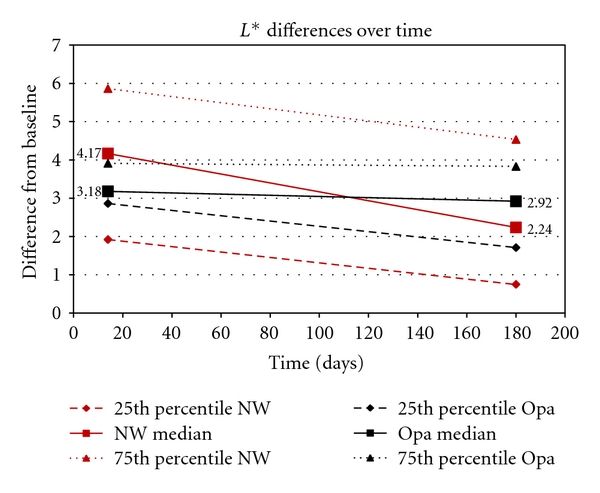
Opalescence (Opa) and Nite White (NW). The 25th percentile, median, and 75th percentile differences in the L* values between the baseline (before treatment) and after treatment (14 days) and between baseline and after 6 months.

**Figure 2:**
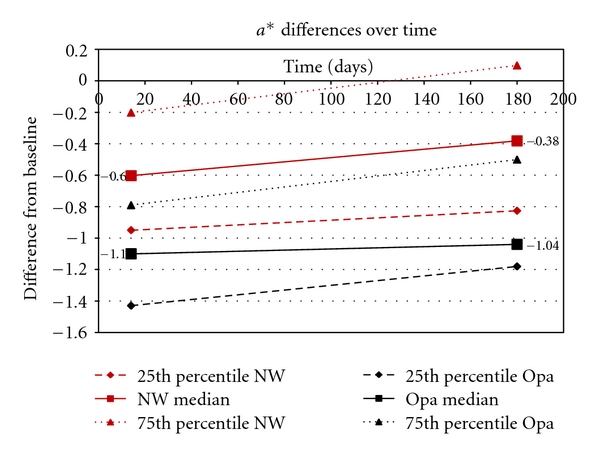
Opalescence (Opa) and Nite White (NW). The 25th percentile, median, and 75th percentile differences in the a* values between the baseline (before treatment) and after treatment (14 days) and between baseline and after 6 months.

**Figure 3:**
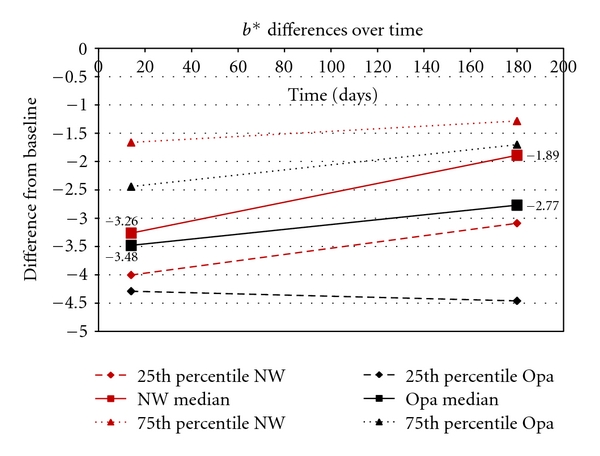
Opalescence (Opa) and Nite White (NW). The 25th percentile, median, and 75th percentile differences in the b * values between the baseline (before treatment) and after treatment (14 days) and between baseline and after 6 months.

**Figure 4:**
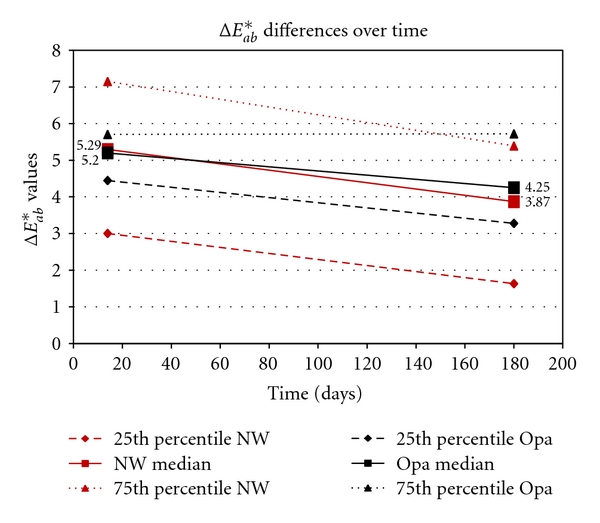
Opalescence (Opa) and Nite White (NW). The 25th percentile, median and 75th percentile differences in the Δ*E*_ab_* values between the baseline (before treatment) and after treatment (14 days) and between baseline and after 6 months.

Figure 1 gives a graph for Opalescence and Nite White for the 25th percentile, median, and 75th percentile differences in the L* values between the baseline (before treatment) and immediately after treatment (14 days), as well as between baseline and after 6 months. Figures 2 and 3 show the same differences for the a* and b* values over time.  Figure 4 shows the total color change (Δ*E*_ab_*) immediately after treatment (14 days) and after 6 months. 

Highly statistically significant differences (*P* < .01; Wilcoxon Signed Rank Sum Test) were found for all components (L*, a*, b*) for both products between baseline values and the values obtained after treatment (14 days later) as well as between baseline and after 6 months (180 days). However, no significant differences (*P* > .05) were found between 14 days and 180 days for the L*, a*, b*, and Δ*E*_ab_* for Opalescence, but for Nite White significant differences (*P* < .05; decrease in color) in L*, a*, b*, and Δ*E*_ab_* were found.

When comparing the two products, no significant differences (*P* > .05) in the L*, b*, and Δ*E*_ab_* were found immediately after treatment (after 14 days) as well as after 180 days. However, the a* values between the two products differed significantly (*P* < .05) after 14 days as well as after 180 days. 

## 4. Discussion

This clinical study employed the spectrophotometer to determine the color change of teeth up to 6 months after treatment. The employed instrument (Model: CM-2600d, Konica Minolta Sensing, Inc., Japan) has diffused illumination with a viewing angle of 8°. The repeatability in terms of the standard deviation when Δ*E*_ab_* is measured is within 0.04, while the interinstrument agreement for the Δ*E*_ab_* measurement is within 0.2 (MAV/SCI). 

The CIE system of color [24] is a mixture of hue (green, red, blue, yellow, etc.), lightness (bright colors and dark colors), and saturation (vivid colors and dull colors). With the spectrophotometer, one can quantify colors by measuring them numerically in a three-dimensional color space (L*a*b*), where the L* value varies from the darkness with a value of 0 to lightness with a value of 100. The values of a* and b* are the chromaticity coordinates. The a* value varies from a negative side (more greenish) to the positive side (more reddish), while the b* value varies from the more blue side (negative side) to the more yellow side (positive side). In this study, the total color (Δ*E*_ab_*) was measured according to the following formula: Δ*E*_ab_* = [(ΔL*)^2^+(Δa*)^2^+(Δb*)^2^]^1/2^, where ΔL*, Δa*, and Δb* are the changes which occurred in these components [25]. Measuring only Δ*E*_ab_* (like with a shade-guide) would mask the information about the spectrum of the tooth colors (Figures 1–3).

The area between the 25th and 75th percentile (Figures 1–4) gives an indication where 50% of the values were. This relatively large spreading of the results showed that teeth from different people bleached differently. The only statistically significant difference (*P* < .05) in the bleaching effects between Nite White and Opalescence were found in the a* values (Figure 2) after 14 days as well as after 6 months where teeth treated with Nite White were found to become more reddish. However, on a 10% significant basis, the b* value of Nite White after 6 months (Figure 3) deteriorated more than that of Opalescence which means the teeth became more yellowish over time. In general, the graphs (Figures 1–4) show that bleaching with Nite White resulted in a less sustainable effect in all components (L*, a*, b*  and  Δ*E*_ab_*) which can clearly be observed after a 6-month period. The median L * value (3.18) decreased (less white/bright) by 8.2% for Opalescence but by 46.3% for Nite White from immediately after treatment (4.17) to 6-months later. The a* value decreased (more reddish) by only 5.5% for Opalescence but by 36.7% for Nite White over 6 months. The b* value also decreased (more yellow) by 20.4% for Opalescence but by 42% for Nite White during the 6 month period after treatment. Thus, for Nite White teeth became less bright/white, more reddish, and more yellow over time than for Opalescence. However, the combined effect of the better initial L* value with the poorer a* value of Nite White in comparison to that of Opalescence resulted in a Δ*E*_ab_* value (5.29) of almost identical to that for Opalescence (5.20). This finding further emphasizes the danger of only determining the total whitening effect like with a shade guide. Nite White shows an overall decrease (Δ*E*_ab_*; Figure 4) after 6 months of 26.8%, while it was less at 18.3% for Opalescence. 

Different methods have been used to determine the color of teeth. Basically it varies between measurements with shade guides, colorimeters, and spectrophotometers. The problem however is that the values obtained with shade guides cannot be compared directly to the others. When shade guide values were arranged with tabs from lighter to darker teeth (B1 to C4) [7, 8, 25] and thereafter the Δ*E*_ab_* value for each tab measured in the same sequence, it became clear that the Δ*E*_ab_* values varied in magnitude but with no definite trend as stated for a shade guide.

Swift et al. [8] found that patients who used 10% carbamide peroxide gel nightly for 2 weeks showed a 7 unit improvement (Vita Lumin shade guide) that decreased with 28% after 6 months which is quite similar to the 27% decrease found for Nite White ACP in this study (Figure 4). Leonard et al. [9] revealed that the whitening effect caused by 10% carbamide (Nite White Classic; 8–10 h/d for 14 days; A3 or darker) reported 5 units lighter teeth after 3.8 years. In three different articles [26–28] on 10% (C1 or darker teeth) carbamide peroxide which was applied for 2 h/day for 3 weeks, a Δ*E*_ab_* value of 4.1 was reported one week after treatment, a value of 3.8 after 6 months and a value of 4.3 after one year. From these Δ*E*_ab_* values, it can be deduced that there was no color relapse even after a one-year period which is in contrast to this study (Figures 1–4). In another study [7] where Opalescence PF 10% was also used overnight, but in contrast to this study applied for 21 days the lightness first increased with 3 units from A2 to A1 with no relapse after 21 days, 30 days, or 180 days. Again the no relapse is in contrast to this study. A study on a 10% carbamide peroxide whitener [25] on A2 or darker teeth when treated overnight (8 hours) for two weeks showed a Δ*E*_ab_* = 6.57 after treatment with a 24% relapse after a 3-month period.

In contrast to our study, Ishikawa-Nagai et al. [19] reported a total higher tooth whitening effect (applied to darker than A2 teeth for 14 days at least for 4 hours daily) for Nite White Excel (5.84) than for Opalescence PF 10% (5.03), while we found no difference between the two but a quite similar value for Opalescence (5.20). 

In a recent study [12] on Opalescence PF a much lower total Δ*E*_ab_* increase of 3.66 was reported in contrast to the 5.2 (Figure 4) found in this study. This difference is most probably due to the color selection of the teeth to be treated. When darker teeth (A2 or darker) were selected for the experiment, the whitening effect was found to be higher (5.2; in this study) than when all teeth were included (3.66). Ritter et al. [10] (treated 6 weeks for about 7 h/day) stated that a 10% carbamide peroxide solution provided patients with aesthetic results up to 12 years post-bleach, while Swift et al. [8] concluded that a 10% carbamide peroxide still had satisfactory results 2 years postbleaching. In a study on 10% carbamide peroxide, it was reported that the whitening effect remained similar after a 6-month period, although the treatment period was different 2 h/day for 3 weeks [26]. Goo et al. [29] (Δ*E*_ab_* = 5.35) (applied 2 weeks for 6 h/day) also found 10% carbamide peroxide effective with a linear increase in the whiteness as the period of treatment increased, while Deliperi and Bardwell [30] reported no decrease of the shade after a 2-year followup with 10% carbamide peroxide.

In the end, the question however is whether the decrease in tooth color found after 6 months (Figures 1–4) in this study is still acceptable or whether it is necessary to re-bleach. The answer to this question might vary from person to person. However, with a relapse of only 26.8% for Nite White ACP and 18.3% for Opalescence PF (Figure 4), found in this study, it would not be advisable to re-bleach after 6 months.

## 5. Conclusions

Opalescence PF 10% as well as Nite White ACP 10% carbamide peroxide are effective at-home bleaching products. There was a significant aesthetic improvement in all 3 color coordinates (L*, a*, and b*) for up to 6 months postbleaching. Nite White showed a higher degree of relapse (27%) than Opalescence (18%) over the 6-month period. It is suggested that rebleaching after 6 months is not necessary.
